# TRPM7 regulates proliferation and polarisation of macrophages

**DOI:** 10.1242/jcs.151068

**Published:** 2014-11-01

**Authors:** Tom Schilling, Francesc Miralles, Claudia Eder

**Affiliations:** 1 Infection and Immunity Research Institute, St George's, University of London, London SW17 0RE, UK; 2 Cardiovascular and Cell Sciences Research Institute, St George's, University of London, London SW17 0RE, UK; 3 Institute for Medical and Biomedical Education, St. George's, University of London, London SW17 0RE, UK

**Keywords:** Ion channel, Macrophage, Proliferation, Polarisation, Transient receptor potential channel, TRP channel

## Abstract

Ion channels play pivotal roles in regulating important functions of macrophages, such as cytokine and chemokine production, migration, proliferation, phagocytosis and others. In this study, we have identified the transient receptor potential cation channel, subfamily M, member 7 (TRPM7) for the first time in macrophages. TRPM7 activity is differentially regulated in macrophages, i.e. current density in TRPM7 is significantly larger in anti-inflammatory M2-type macrophages than in untreated and in pro-inflammatory M1-type macrophages, whereas mRNA levels of *TRPM7* remain unchanged upon cell polarisation. The specific TRPM7 inhibitors NS8593 and FTY720 abolish proliferation of macrophages induced by interleukin-4 (IL-4) and macrophage colony-stimulating factor (M-CSF), respectively, whereas proliferation arrest was not accompanied by induction of apoptosis or necrosis in macrophages. Furthermore, NS8593 and FTY720 prevented polarisation of macrophages towards the anti-inflammatory M2 phenotype. Inhibition of TRPM7 reduced IL-4-induced upregulation of arginase-1 (*Arg1*) mRNA levels and Arg1 activity, and abolished the inhibitory effects of IL-4 or M-CSF on LPS-induced TNF-α production by macrophages. In summary, our data suggest a main role of TRPM7 in the regulation of macrophage proliferation and polarisation.

## INTRODUCTION

Macrophages play important roles in health and disease. Their functions include immune surveillance, bacterial killing, tissue remodelling and repair, clearance of cell debris and more (Gordon and Taylor, 2005; Martinez et al., 2009; Pollard, 2009; Wynn et al., 2013). Macrophages can have beneficial as well as detrimental effects for the outcome of several diseases depending on the microenvironment and activation state of the cell. A general classification of activated macrophages into M1-type ‘classically activated’ macrophages and M2-type ‘alternatively activated’ macrophages has been proposed recently (Martinez et al., 2009; Sica and Mantovani, 2012; Wynn et al., 2013). M1-type macrophages exert antimicrobial actions and promote inflammatory processes, whereas M2-type macrophages are involved in the resolution of inflammation and promote tissue repair mechanisms (Martinez et al., 2009; Sica and Mantovani, 2012; Wynn et al., 2013). Although several factors have been identified causing the transformation, i.e. polarisation, of macrophages towards the M1 or M2 phenotype, underlying mechanisms are poorly understood.

Over the past few years, there has been an increasing interest in ion channels of macrophages, in particular in transient receptor potential (TRP) channels, as they play major roles in controlling macrophage functions, such as phagocytosis (Link et al., 2010), production of chemokines and cytokines (Yamamoto et al., 2008; Knowles et al., 2011), cell survival (Serafini et al., 2012). Thus, they provide potential therapeutic targets in a variety of diseases (Eder, 2010). The role of ion channels in regulating polarisation of macrophages has not been addressed to date.

TRPM7 has recently been identified in a variety of tissues and cell types, where it can have both ion channel and kinase activity. TRPM7 is permeable to Mg^2+^ and Ca^2+^ and, thus, regulates intracellular ion concentrations, and its protein kinase domain mediates autophosphorylation and phosphorylates serine/threonine residues. By regulating Mg^2+^ homeostasis in cells, TRPM7 has been found to affect cellular growth and differentiation (Harteneck, 2005; Paravicini et al., 2012). Here, we have identified TRPM7 in macrophages, where it is involved in the polarisation of macrophages towards the M2 phenotype as well as in proliferation of M2 macrophages.

## RESULTS AND DISCUSSION

### Functional TRPM7 channels in macrophages

**Fig. 1.**
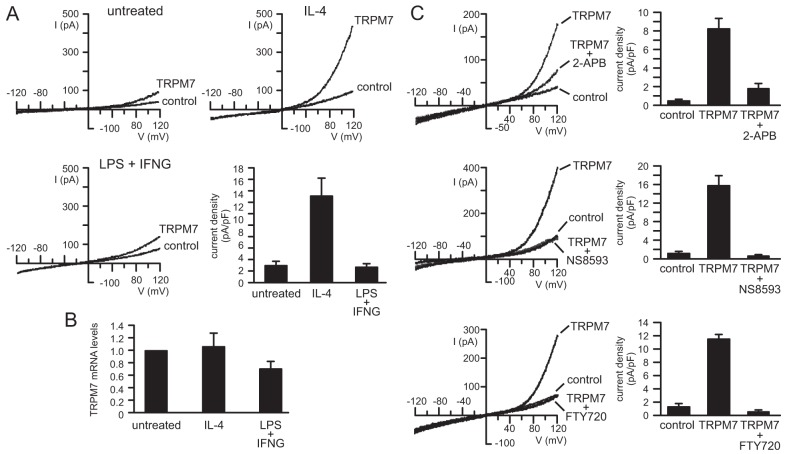
**TRPM7 expression and currents in macrophages.** (A,C) Macrophages were clamped at a holding potential of −30 mV and voltage ramps were applied from −120 mV to +120 mV for a duration of 480 ms every 20 s. (A) Current traces were recorded before (control) and after full activation of TRPM7 currents (TRPM7). Mean current densities (in pA/pF) of leak-subtracted TRPM7 currents were determined at +120 mV in untreated macrophages (*n* = 26) and in macrophages stimulated with 20 ng/ml IL-4 (IL-4; *n* = 13) or 1 µg/ml LPS and 10 ng/ml IFN-γ (LPS+IFNG; *n* = 16). (B) TRPM7 mRNA levels of macrophages kept untreated or stimulated with 20 ng/ml IL-4 or 1 µg/ml LPS and 10 ng/ml IFN-γ were determined by qPCR. (C) Current traces and corresponding current densities of cells recorded before (control) and during activation of TRPM7 channels (TRPM7) and during superfusion of cells with 500 µM 2-APB (*n* = 5), 50 µM NS8593 (*n* = 10) or 3 µM FTY720 (*n* = 5) following TRPM7 current activation.

In a first set of experiments, ion channel expression and activity were examined in macrophages of different activation states, for which cells were activated either with both lipopolysaccharide (LPS) and interferon-γ (IFN-γ, also known as INFG) or with interleukin-4 (IL-4, also known as IL4). Previous *in vitro* experiments have revealed that pro-inflammatory M1-type macrophages can be induced by combined application of LPS and IFN-γ, whereas anti-inflammatory M2-type macrophages can be induced by IL-4 (Martinez et al., 2009; Sica and Mantovani, 2012; Davis, 2013; McWhorter et al., 2013; Wynn et al., 2013). Functional ion channels were identified using the patch clamp technique. The most striking difference between untreated and IL-4-stimulated M2 macrophages was found to be the activity of TRPM7, which was increased significantly in response to stimulation with IL-4. To evoke TRPM7-mediated currents in macrophages, whole-cell patch clamp experiments were performed by using Mg^2+^-free pipette solution. TRPM7 currents elicited within seconds and increased in size gradually with time, whereas stable current amplitudes were reached after 3−5 minutes. Fig. 1A shows typical examples of TRPM7 currents in untreated cells, and in cells treated with IL-4 and LPS+IFN-γ. The mean TRPM7 current density of IL-4-treated macrophages (13.6±2.9 pA/pF; *n* = 13 cells) was 4.7-fold higher (*P*<0.001) than that of untreated macrophages (2.9±0.5 pA/pF; *n* = 26 cells). In contrast, shifting macrophages into the pro-inflammatory M1 phenotype with LPS and IFN-γ did not significantly affect TRPM7 current density (2.6±0.5 pA/pF; *n* = 16 cells; *P* = 0.999) of macrophages. Quantitative RT-PCR experiments revealed no significant differences in expression levels of *TRPM7* mRNA between untreated macrophages and those treated with either IL-4 (*P* = 0.999) or LPS+IFN-γ (*P* = 0.971) (Fig. 1B). TRPM7 currents of macrophages were inhibited by the general TRP channel inhibitor 2-APB, and by the TRPM7-specific inhibitors NS8593 (Chubanov et al., 2012) and FTY720 (Qin et al., 2013), as demonstrated in Fig. 1C.

### Effects of TRPM7 inhibition on proliferation of macrophages

**Fig. 2.**
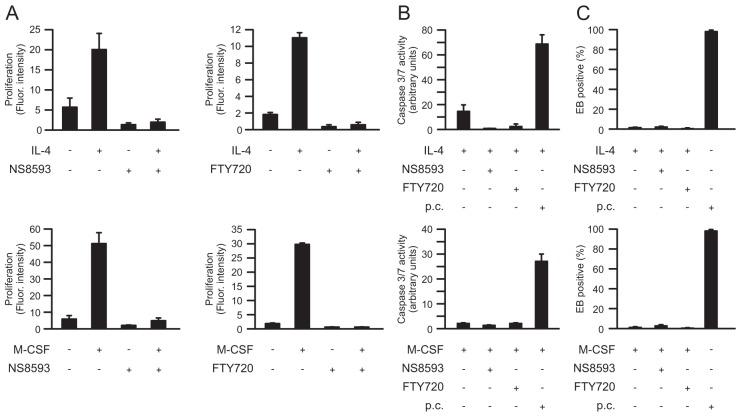
**Inhibitory effects of TRPM7 inhibitors on macrophage proliferation.** (A) Proliferation of macrophages was induced by either IL-4 or M-CSF, in absence or presence of TRPM7 blockers as indicated. (B) No activity of caspase 3/7 was found in macrophages treated with IL-4 or M-CSF in absence or presence of NS8593 or FTY720, indicating absence of apoptosis. As positive control (p.c.), macrophages were exposed to 5 µM staurosporine for 24 h in each experimental condition. (C) TRPM7 inhibitors have no effect on macrophage necrosis determined as percentage of ethidium bromide (EB)-positive cells. As positive control (p.c.), cells were damaged by freezing and subsequent thawing. (A-C) Macrophages were kept untreated or were treated with 20 ng/ml IL-4 and/or 50 ng/ml M-CSF in absence or presence of 50 µM NS8593 or 3 µM FTY720, as indicated.

The striking increases in the current density of TRPM7 within IL-4-stimulated macrophages led us further to investigate the functional importance of TRPM7 in these macrophages. First, we tested whether IL-4-induced proliferation of macrophages was affected by TRPM7 inhibitors. IL-4 has recently been identified as potent stimulator of macrophage proliferation (Jenkins et al., 2011). Compared with untreated macrophages, the proliferation rate of macrophages treated with IL-4 for 3 days (*n* = 27 experiments; *P*<0.001) increased 4.8 fold. This IL-4-induced proliferation of macrophages was completely inhibited when TRPM7 was blocked with NS8593 (*n* = 9 experiments) or FTY720 (*n* = 9 experiments), as demonstrated in Fig. 2A, top row.

To determine whether TRPM7 activity is required exclusively for IL-4-induced macrophage proliferation or whether it is also involved in macrophage proliferation induced by other stimuli, further experiments were performed on macrophages stimulated with macrophage colony-stimulating factor (M-CSF, also known as CSF1) – a well-known inducer of macrophage proliferation (Stanley et al., 1976). M-CSF induced a 13-fold increase in macrophage proliferation rate (*n* = 39 experiments; *P*<0.001). Intriguingly, the proliferation rate of macrophages treated simultaneously with M-CSF and IL-4 was 43% lower (*n* = 12 experiments; *P*<0.001) than that determined for macrophages treated with M-CSF alone (data not shown). These data are in agreement with Arpa and colleagues, who demonstrated an inhibition of M-CSF-stimulated proliferation by IL-4 due to induction of p21^Waf1^ (Arpa et al., 2009). Similar to observations made on IL-4-stimulated macrophages, M-CSF-induced proliferation of macrophages was abolished by NS8593 (*n* = 9 experiments) or FTY720 (*n* = 9 experiments) (Fig. 2A, bottom row).

Next we tested whether the inhibitory effects of TRPM7 inhibitors on proliferation of macrophages were due to or accompanied by cell death. To investigate cell apoptosis, the combined activity of caspases 3 and 7 (caspase 3/7) – indicators of apoptosis – was determined. As demonstrated, neither NS8593 (*n* = 9 experiments in each case) nor FTY720 (*n* = 9 experiments in each case) induced caspase-3/7 activity in macrophages that are treated with IL-4 (Fig. 2B, top) or M-CSF (Fig. 2B, bottom). To investigate cell necrosis, cells were stained with ethidium bromide. As demonstrated in Fig. 2C, macrophages cultured in the presence of NS8593 or FTY720 did not stain positive for ethidium bromide (*n* = 3 experiments per experimental condition). Thus, it can be excluded that the NS8593- or FTY720-induced inhibition of macrophage proliferation was caused or accompanied by apoptotic or necrotic cell death.

### Effects of TRPM7 inhibition on macrophage polarisation

**Fig. 3.**
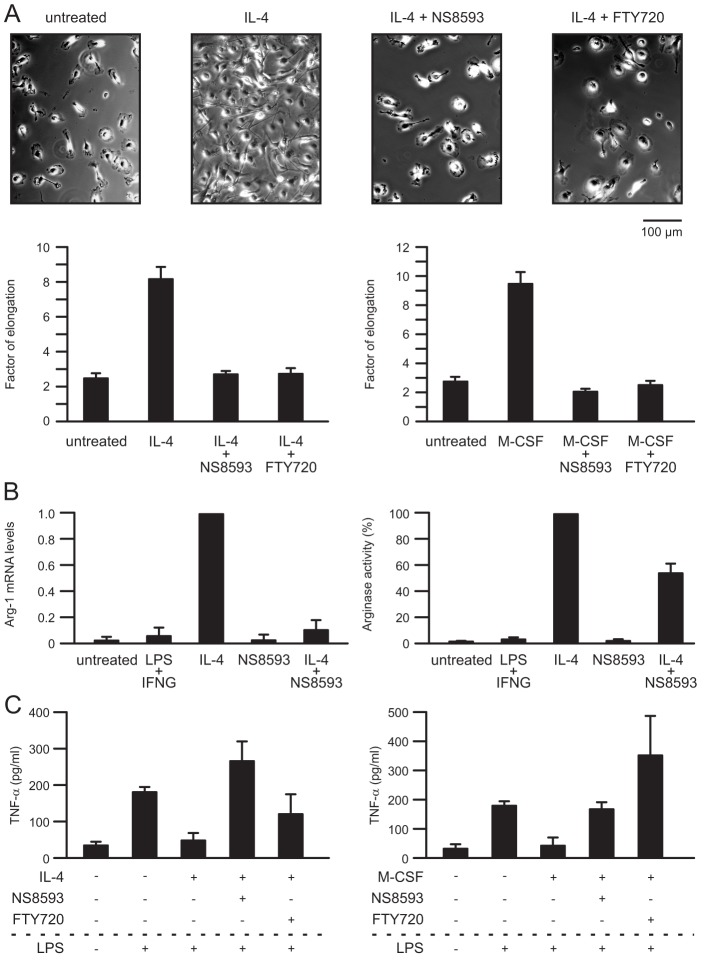
**Inhibitory effects of TRPM7 inhibitors on macrophage polarisation.** (A) Micrographs show examples of brightfield DIC of IL-4-treated macrophages in the absence and presence of TRPM7 inhibitors. Graphs show the factor of elongation for untreated macrophages, or macrophages treated with IL-4 or M-CSF with or without additional treatment of TRPM7 inhibitors as indicated. (B) Inhibitory effects of NS8593 on *Arg1* expression and Arg1 activity of IL-4-treated macrophages. Normalised *Arg1* mRNA levels (left) and Arg1 activity (right) determined in macrophages kept untreated or stimulated with LPS/IFN-γ or IL-4 in absence or presence of NS8593 as indicated. (C) TNF-α release from macrophages stimulated with 1 µg/ml LPS for 4 h. Prior to LPS stimulation, macrophages had not been cultured as normal (untreated) or had been pre-treated with IL-4 in absence or presence of NS8593 or FTY720 as indicated. (A-C) Macrophages were cultured with or without additional treatment of 20 ng/ml IL-4 or 50 ng/ml M-CSF in absence or presence of 50 µM NS8593 or 3 µM FTY720 for 3 days as indicated.

In another series of experiments we asked whether TRPM7 activity is required for the transformation of IL-4-stimulated macrophages into the M2 phenotype. First, changes in cell morphology were evaluated. It has recently been demonstrated that polarisation of macrophages towards the M2 phenotype is associated with an elongated cell shape, and that it is sufficient to prevent macrophage elongation in order to inhibit full transformation of macrophages to the M2 phenotype (McWhorter et al., 2013). Untreated macrophages exhibited a roundish morphology with – in some cases – short processes (Fig. 3A, first micrograph). In contrast, macrophages treated with IL-4 (Fig. 3A, second micrograph) and/or M-CSF exhibited an elongated morphology as described by McWhorter et al., 2013. Combined treatment with IL-4 and M-CSF induced a 2.2-fold (*P*<0.01) increase in the cells' elongation factor (not shown), whereas the factor of elongation of macrophages that had been exposed to either IL-4 or M-CSF increased 3.5-fold (*P*<0.001 in both cases) (Fig. 3A). In contrast, morphological changes did not occur in macrophages that were treated with NS8593 or FTY720 as well as IL-4- or M-CSF. Therefore, the factor of elongation remained unchanged (Fig. 3A).

Because upregulation of arginase-1 (Arg1) has been identified as a typical marker of M2-type macrophages (Sica and Mantovani, 2012), we next tested whether *Arg1* mRNA expression levels and Arg1 activity were affected by inhibition of TRPM7 in macrophages. Both *Arg1* mRNA expression levels and Arg1 activity were almost undetectable in untreated macrophages and in LPS+IFN-γ-stimulated M1-type macrophages (Fig. 3B), whereas exposure of macrophages to IL-4 caused substantial increases in *Arg1* mRNA levels and Arg1 activity (*P*<0.001 in both cases) (Fig. 3B). In the presence of NS8593, *Arg1* mRNA levels and Arg1 activity of IL-4 stimulated macrophages were reduced by 89.5% (*n* = 8 experiments; *P*<0.001) and 45.4% (*n* = 6 experiments; *P*<0.01), respectively (Fig. 3B).

To further test whether blocking of TRPM7 activity affects macrophage polarisation, LPS-induced release of TNF-α was determined. In control cells that had been cultured without any pre-treatment, stimulation with LPS caused substantial (*n* = 4 experiments; *P*<0.05) release of TNF-α (Fig. 3C, left graph, second column). In contrast, M2-type macrophages pre-treated with IL-4 for 3 days failed to produce TNF-α following subsequent stimulation with LPS. However, TNF-α production could be induced by LPS in macrophages pre-treated with IL-4 in the presence of TRPM7 inhibitors (Fig. 3C). TNF-α production by LPS-stimulated macrophages pre-treated with IL-4 in the presence of TRPM7 inhibitors did not differ significantly (*n* = 3 experiments, *P* = 0.377 for NS8593; *n* = 3 experiments, *P* = 0.654 for FTY720) from that of LPS-stimulated control macrophages. Similarly, macrophages pre-exposed to M-CSF – which can also induce the anti-inflammatory M2-like phenotype in macrophages (Lacey et al., 2012) – were unable to produce significant levels of TNF-α in response to stimulations with LPS, whereas levels of TNF-α increased in LPS-stimulated macrophages when TRPM7 was inhibited by NS8593 or FTY720 (Fig. 3C).

### Conclusions

This is the first study to demonstrate presence, regulation and functional importance of TRPM7 in macrophages. We have identified two main functions of TRPM7 in macrophages: (1) their role in the regulation of macrophage proliferation and (2) their requirement for the polarisation of M2-type macrophages. Our data suggest that proliferating M2-type macrophages are characterised by enhanced TRPM7 activity, which is required for optimal proliferation of macrophages, independently of the stimulus causing macrophage proliferation. To our knowledge, this is the first description of functional TRPM7 in macrophages. Although mRNA expression levels of *TRPM7* remained unchanged, the TRPM7 current density was increased following treatment of macrophages with IL-4. These data suggest that IL-4 does not affect TRPM7 expression, but rather modulates the activity of this cation channel, possibly regulating the influx of Mg^2+^ and Ca^2+^ at physiological membrane potentials (Harteneck, 2005; Paravicini et al., 2012). Both, Mg^2+^ and Ca^2+^ are essential for optimal proliferation of other cell types (Wolf et al., 2008; Machaca, 2011). It has been suggested that Mg^2+^ is implicated in several processes, e.g. gene transcription and protein synthesis, DNA duplication, and cytoskeletal rearrangement (Wolf et al., 2008). Ca^2+^ regulates a wide variety of cell functions, modulates intracellular signalling pathways, regulates gene expression and has a role during various stages of the cell cycle (Machaca, 2011). Owing to the variety of Mg^2+^- and Ca^2+^-regulated cell processes, the precise role of Mg^2+^ and/or Ca^2+^ in cell cycle progression in macrophages that have been stimulated with IL-4- and M-CSF remains to be elucidated.

In addition to the importance of TRPM7 in macrophage proliferation, we established that TRPM7 has a role in regulating the functional state of macrophages. TRPM7 blockers can inhibit IL-4- or M-CSF-induced changes of cell morphology, upregulation of *Arg1* mRNA expression and upregulation of Arg1 activity. Furthermore, TRPM7 inhibitors prevent the inhibitory effect that IL-4 or M-CSF have on the production of the pro-inflammatory cytokine TNF-α. Together, these data suggest that TRPM7 activity is required for polarisation of macrophages towards the anti-inflammatory M2 phenotype. To our knowledge, this is the first study that investigated the role of an ion channel in the regulation of macrophage polarisation. Why is TRPM7 activity required for the polarisation of macrophages? To date it can only be speculated regarding the importance of TRPM7 activity for the transformation of macrophages into the M2 phenotype. It has been found recently that inhibition of TRPM7 decreases phosphorylation of PI3K and ERK1 and ERK2 (MAPK3 and MAPK1, respectively) in hepatic stellate cells (Fang et al., 2013). Since PI3K/ERK phosphorylation appears to be required for polarisation of macrophages towards the M2 phenotype (Zhang et al., 2011), it is possible that, in macrophages, TRPM7 activity modulates intracellular pathways, such as PI3K/AKT/ERK signalling, that are involved in M2 macrophage polarisation. Further experiments are required to elucidate the precise mechanism by which inhibition of TRPM7 affects macrophage polarisation towards the M2 phenotype.

## MATERIALS AND METHODS

### Macrophage cultures

C57Bl/6 mice were supplied by Charles River (Margate, UK). Bone-marrow-derived macrophages were prepared as described previously (Eder and Fischer, 1997) with minor modifications. Briefly, bone marrow cells, flushed from femur and tibia with ice-cold PBS, were resuspended for 2 minutes in 155 mM NH_4_Cl solution to lyse erythrocytes. The remaining leukocytes were seeded at 6×10^6^ cells/10 ml in uncoated Petri dishes. Culture medium was Dulbecco's modified Eagle medium (DMEM; LifeTechnologies, Paisley, UK) containing additionally 2 mM L-glutamine and 10% heat-inactivated fetal calf serum (FCS) (LifeTechnologies). The culture medium was changed on days 2 and 5. To support outgrowth of macrophages, the medium was supplemented with 30% supernatant of L-929 fibroblast cultures as a source of M-CSF. On day 8, adherent cells were harvested and subcultured in DMEM/10%FCS (LifeTechnologies) for experiments. Macrophages were maintained for 3 days in the absence or presence of 20 ng/ml IL-4 and/or 50 ng/ml M-CSF with or without 50 µM NS8593 or 3 µM FTY720 as indicated. In some cases, macrophages were cultured for 1 day in DMEM/10%FCS containing 1 µg/ml LPS and 10 ng/ml IFN-γ. This study was performed in accordance with the Animals (Scientific Procedures) Act 1986 under regulations from the Home Office England.

### Chemicals

The following drugs were used in this study: IL-4, M-CSF, IFN-γ (all from R&D systems, Abingdon, UK), LPS, 2-APB, staurosporine, N-[(1R)-1,2,3,4-tetrahydro-1-naphthalenyl]-1H-benzimidazol-2-amine hydrochloride (NS8593) (all from Sigma-Aldrich, Dorset, UK), FTY720 hydrochloride (FTY720; Cayman Europe, Tallin, Estonia).

### Electrophysiological recordings

For patch clamp experiments, 2×10^5^ cells per well were seeded on uncoated glass coverslips in 24-well tissue culture plates and cultured as indicated. Whole-cell membrane currents were measured using a patch clamp recording system as described previously (Schilling and Eder, 2009). The intracellular solution (pH 7.3) contained: 20 mM NaCl, 100 mM Na-D-gluconate, 1 mM CaCl_2_, 10 mM HEPES, 11 mM EGTA. The extracellular solution (pH 7.4) contained: 135 mM NaCl, 2 mM CaCl_2_, 1 mM MgCl_2_, 10 mM HEPES, 10 mM D-glucose.

### Proliferation assay

To determine proliferation rates, 1×10^4^ cells per well were plated in black 96-well clear, flat-bottomed tissue culture plates and cultured as indicated. Proliferation of macrophages was determined by CyQUANT proliferation assay (LifeTechnologies) according to the manufacturer's instructions. Fluorescence intensity for each well was measured using a microplate reader (FLUOstar Omega, BMG Labtech, Aylesbury, UK) equipped with a 500±10 nm excitation filter and a 530±10 nm emission filter. Background-corrected data were collected in at least three independent experiments from at least three different wells.

### Apoptosis assay

To determine apoptosis, 1×10^4^ cells per well were seeded in black 96-well plates and cultured as indicated. Activity of caspases 3 and 7 (caspase 3/7) as a measure of cell apoptosis was assessed using CellEvent Caspase-3/7 Green Detection Reagent (LifeTechnologies) according to the manufacturer's instructions. Fluorescence intensity of cells was detected using a microplate reader (FLUOstar Omega, BMG Labtech), which was equipped with a 500±10 nm excitation and a 530±10 nm emission filter. Background-corrected data were collected in at least three independent experiments from at least three wells. All data were normalised to the mean cell count determined for each experimental condition by the CyQUANT proliferation assay.

### Necrosis assay

Membrane integrity of macrophages was investigated using ethidium bromide (LifeTechnologies) staining as described previously (Schilling et al., 2004). Images of at least three different visual fields for at least three independent experiments per condition were collected and analysed.

### Analysis of cell morphology

To quantify changes in macrophage morphology, the factor of elongation was determined as described previously (McWhorter et al., 2013). In brief, macrophages were plated at a density of 1.5×10^5^ cells on glass coverslips in 24-well plates and cultured as indicated. Subsequently, cells were fixed for 5 min with 2% paraformaldehyde and 0.2% glutaraldehyde. After washing cells twice with PBS, brightfield pictures were taken and analysed using the programme ImageJ (NIH, Bethesda, MA). The factor of elongation was determined for each individual macrophage by dividing the length of the longest axis by the length of the shortest axis across the cell nucleus.

### Arginase assay

To determine Arg1 activity, 1×10^6^ cells per well were plated in six-well tissue culture dishes and cultured as indicated. Subsequently, Arg1 activity in cell lysates and culture supernatants was assayed using QuantiChrom Arginase Assay kit (BioAssay Systems, Hayward, CA) according to the manufacturer's instructions. For each experimental condition, Arg1 activity was normalised to protein concentration, which was determined from cell pellets using a Micro BCA protein assay kit (ThermoScientific, Rockford, IL) according to the manufacturer's protocol.

### Detection of TNF-α by ELISA

Macrophages were plated in six-well culture dishes at a density of 1×10^6^ cells per well and cultured as indicated. Thereafter, cell supernatants were collected and stored at −80°C. To determine concentrations of tumour necrosis factor alpha (TNF-α) in supernatants of macrophages, murine TNF-α Quantikine Immunoassay kit (R&D systems, Abingdon, UK) was used according to the manufacturer's instructions. The sensitivity of the TNF-α sandwich ELISA kit was 5 pg/ml.

### Quantitative RT-PCR

RNA was purified with the E.Z.N.A Total RNA kit (Omega Bio-tek, Norcross, GA). RNA was DNase I treated in-column during the purification process and 500 ng RNA were reverse transcribed using random hexamers and Maxima reverse transcriptase according to the manufacturer's instructions (Fisher Scientific, Hampton, USA). Quantitative PCR was conducted on a C1000 Thermal Cycler (Biorad, Hercules, CA) with 30 ng of reverse transcribed RNA and DyNAmo Flash SYBR Green qPCR mix (Thermo Scientific, Walham, MA) using the following mouse-specific primers: Arg-1, forward: 5′-CTCCAAGCCAAAGTCCTTAGAG-3′; Reverse, 5′-AGGAGCTGTCATTAGGGACATC-3′), TRPM7, forward: 5′-AGGATGTCAGATTTGTCAGCAA-3′; reverse: 5′-CCTGGTTAAAGTGTTCACCCAA-3′). Primers for the ribosomal protein L7 RNA were used as a control and have been described previously (Perdiguero et al., 2007).

### Statistics

All data are presented as mean values ± standard error of the mean (±s.e.m.) and numbers of analysed samples are indicated. The statistical significance of differences between experimental groups was evaluated either by paired *t*-tests (analysis of drug effects on TRPM7 currents) or by one-way ANOVA (all other experiments) using SPSSv19. Tukey's test was used for post hoc comparison after confirming homogeneity of variances with Levene's test. Data were considered to be statistically significant with *P*<0.05.
